# Descriptive analysis of colour Doppler echocardiography results in neonates with congenital heart disease in eastern China: a retrospective cohort study

**DOI:** 10.3389/fped.2025.1638808

**Published:** 2025-07-31

**Authors:** Hui Wang, Qifeng Guo, Xiuhua Yu

**Affiliations:** ^1^ Department of Ultrasound, No.903 Hospital of PLA Joint Logistic Support Force, Hangzhou, China; ^2^ Department of Neurology, No.903 Hospital of PLA Joint Logistic Support Force, Hangzhou, China

**Keywords:** congenital heart disease, colour Doppler echocardiography, neonate, patent foramen ovale, patent ductus arteriosus, ventricular septal defect

## Abstract

**Introduction:**

Colour Doppler echocardiography (Echo) is the preferred examination method for cardiac health in newborns. Since 2019, an neonatal echocardiographic screening program has been conducted for all newborns in Hangzhou, China. Herein, we conducted a descriptive analysis of all newborns screened at our hospital, aiming to explore the prevalence in neonatal Echo abnormality.

**Methods:**

The data of 813 neonates who underwent Echo between 2019 and 2024 were retrospectively analysed. Subsequently, we compared the differences in clinical data between neonates with positive (positive neonates) and negative (negative neonates) screening results and further analysed the correlation between Echo results and gestational age at delivery, maternal age, sex, and weight.

**Results:**

The overall prevalence of neonatal Echo abnormality was 83.8%. No significant differences in the clinical data were noted between positive and negative neonates. Multivariate logistic regression analysis subsequently revealed that only gestational age at delivery was an independent influencing factor for Echo abnormality [odds ratio (OR) = 0.813, *P* < 0.05]. Neonatal birth weight was found to be an independent influencing factor for patent ductus arteriosus (OR = 1.001, *P* < 0.05), whereas neonatal sex, gestational age at delivery, and maternal age were not identified as independent influencing factors for certain abnormalities.

**Discussion:**

This study highlights the importance of early pregnancy detection through timely pregnancy testing, facilitating early prenatal care for women and thereby reducing the risk of premature birth and low birth weight. Neonatal Echo screening holds substantial value in early detection, enabling the monitoring of cardiac abnormalities and facilitating the provision of early intervention measures.

## 1 Introduction

Congenital heart disease (CHD) is caused by structural abnormalities in the heart and large blood vessels resulting from a developmental disorder or abnormality during embryonic development or closure failure of a normal fetal circulatory pathway. CHD is the most common congenital malformation (1). The World Health Organization (2) has indicated that approximately 1.5 million perinatal infants worldwide are born with CHD each year. Epidemiological data have further shown that CHD is present in 4%–10% of live births (2). Although present at birth, some newborns with CHD may initially be clinically asymptomatic, only developing symptoms later in life (3). Delayed diagnosis can lead to a considerable increase in morbidity and mortality (4, 5), highlighting the importance of understanding the incidence, early diagnosis, and timely treatment of CHD (6). Routine screening, timely diagnosis, rapid clarification of the specific types of neonatal heart disease, and early treatment can reduce mortality and improve patients’ quality of life (7).

Clinicians may rely on clinical manifestations and symptoms in newborns to judge the severity of CHD; however, detection of cardiac abnormalities with mild clinical symptoms can be challenging, resulting in missed diagnosis. Following advancements in ultrasound and imaging technology, echocardiography (Echo), cardiac magnetic resonance imaging, and computed tomography are being increasingly applied in the diagnosis of cardiac abnormalities. Among these modalities, Echo is the preferred choice for examining the heart status in newborns and infants, due to its efficacy in examining cardiac tissue structure and function and macrovascular connection, which facilitates the early detection of CHD (8). The use of Echo in neonatal CHD screening can reduce the rate of missed diagnosis (9), making it one of the most widely used methods for diagnosing and screening diseases in clinical practice (10). Following advancements in China's current medical technology, colour Doppler ultrasonography has been widely applied in clinical practice for the diagnosis of CHD, particularly of neonatal heart disease, providing clinical advantages in the form of scientific data for early diagnosis and treatment of neonates. Since 2019, Echo screening has been initiated for all newborns in Hangzhou City, Zhejiang Province, China, with our hospital in Hangzhou participating in this screening project. Herein, we conducted a descriptive analysis of all newborns screened at our hospital, aiming to explore the neonatal Echo abnormality.

## 2 Materials and methods

### 2.1 Study design

This retrospective cohort study was conducted at the non-specialist tertiary Joint Service Support Force 903 Hospital located in the eastern city of Hangzhou, China. We collected Echo results from all newborns born at the hospital between October 2019 and February 2024. All 817 newborns born within this time frame were automatically enrolled in the study, which was conducted in accordance with the principles of the Declaration of Helsinki and approved by the Medical Ethics Committee of the Joint Service Support Force 903 Hospital (Approval number: 20230614/02/01/008). Due to the retrospective nature of the study, informed consent was not required.

### 2.2 Cardiac ultrasound examination method for neonates

All newborns were examined in the supine position, with assistance provided as needed, and the examination was performed in a quiet state; in some cases, chloral hydrate sedation was required to prevent neonatal crying from affecting the examination. All available surfaces of the ultrasonic machine were disinfected before and after each examination to ensure that the newborn was protected from infection. Ultrasonic testing was further performed using the IU-22 or IE-33 ultrasound system (Philips Healthcare, Eindhoven, Netherlands) at an operating frequency of 2.5–6.0 MHz. First, the neonates’ cardiac morphology, function, and structure were observed using two-dimensional and M-mode Echo, while relevant parameters, such as ventricular size, were measured. Subsequently, cardiac haemodynamics were recorded using the colour and pulse Doppler modes. The examined section included the parasternal left ventricular long axis, short axis of the heart base, apical four-chamber heart, apical five-chamber heart, four-chamber heart under the xiphoid process, two-chamber heart, and long and short axis of the suprasternal fossa aortic arch. The inner diameter of the aortic sinus, opening of the aortic and mitral valve, thicknesses of the interventricular septum and the posterior wall of the left ventricle, and inner diameters of the pulmonary artery and chamber were all measured. Using the segmental analysis method of the heart and great vessels, we assessed the heart's location, its morphological structure, the connection between the atria and ventricles, the connection between the heart and great vessels, the continuity of the atrioventricular septum, and the morphological structure and function of each of the valves. The diameter of the great vessels, size of the heart chamber, amplitude of motion, thickness of the ventricular wall, and cardiac function of the newborns were also assessed. Concurrently, colour and spectral Doppler were applied to determine the presence of shunts, reflux, and stenosis in the heart chamber, or between the great arteries, and the corresponding results were recorded. The section images and standard section images, including those of the lesion site when applicable, were stored in the ultrasound imaging workstation for further analysis and reference. If a lesion was identified, the corresponding image was stored alongside the standard section image for comprehensive documentation. Three neonatologists in Echo conducted all procedures at our hospital. When in doubt, the paediatric cardiologists were consulted for guidance.

### 2.3 Definitions

Patent foramen ovale (PFO) is defined as a cardiac malformation in which the foramen ovale valve fails to fully close after birth, resulting in a horizontal atrial shunt. Patent ductus arteriosus (PDA) is defined as a high-speed biphasic shunt from the descending aorta to the pulmonary artery, as displayed on colour Doppler. Ventricular septal defect (VSD) is defined as the underdevelopment of the ventricular septum during embryonic development, resulting in an abnormal blood flow and left to right shunting at the ventricular level. Positive (positive neonates) is defined as neonates with positive Echo results, and negative (negative neonates) is defined as neonates with negative Echo results.

### 2.4 Other data collection methods in newborns

After excluding four newborns with incomplete ultrasound results or missing clinical data, demographic data, such as neonatal sex, birth weight, gestational age at delivery, and maternal age, were collected from the medical record system for all remaining participants.

### 2.5 Statistical analysis

Count data are expressed as the number of cases. Measurement data were analysed using the Shapiro–Wilk and Kolmogorov tests for normality, and non-normally distributed data are expressed as the median and quartile spacing. The rank sum or chi-square tests were applied to compare neonates with positive (positive neonates) and negative (negative neonates) screening results for certain Echo abnormalities. Multivariate logistic regression analysis was performed to select independent influencing factors for each Echo abnormality according to the likelihood ratio test (*P* < 0.05). Finally, the regression coefficient, odds ratio (OR), and 95% confidence interval (CI) were calculated, with the 95% CI used as the interval estimation. Data were analysed using SPSS (version 25.0; IBM Corp., Armonk, NY, USA). Statistical significance was set at *P* < 0.05.

## 3 Results

### 3.1 Demographic characteristics

A total of 813 neonates, including 415 male and 398 female individuals, were retrospectively included. Most neonates underwent Echo before discharge, and the average age at Echo screening was 3.82 ± 1.43 days. In this cohort, 132 neonates had negative screening results, 505 had only one abnormality, 150 had two abnormalities, and 26 had three or more abnormalities (including a case of complex CHD). The overall prevalence of neonatal Echo abnormality was 83.8%. Table 1 presents the demographic and clinical data, such as sex, weight, age, gestational age at delivery, and maternal age, of all newborns.

**Table 1:** Clinical data of the neonates.

Characteristic	Positive group			Negative group
	One abnormality	Two abnormalities	Three or more abnormalities	
Sex, *n* (%)				
Male	255 (50.5)	70 (46.7)	18 (69.2)	72 (54.5)
Female	250 (49.5)	80 (53.3)	8 (30.8)	60 (45.5)
Age, median (IQR, days)	4 (1)	4 (1)	3 (1)	4 (2)
Birth weight (g)	3,220 (545)	3,342.5 (543)	3,220 (551)	3,317.5 (488)
Gestational age (weeks)	39 (2)	39 (2)	38.5 (2)	39 (2)
Maternal age, median (IQR, years)	31 (6)	30 (5)	29.5 (6)	30 (5)

IQR, interquartile range.

### 3.2 Comparison of data between the positive and negative neonates

According to the normality results on the Shapiro–Wilk and Kolmogorov tests, some data did not conform to normal distribution; therefore, a rank sum test was used to compare quantitative data between the groups. No significant differences were observed between the data of the positive and negative neonates (all *P* > 0.05). The data of the two groups are presented in Table 2. Multivariate logistic regression analysis was applied to screen whether newborns’ age and weight, gestational age, and maternal age were independent influencing factors for Echo abnormalities (*P* < 0.05). The results confirmed gestational age as an independent influencing factor of Echo abnormalities (OR = 0.813, *P* < 0.05), while no other indicators showed significance (all *P* > 0.05) (Table 3).

**Table 2:** Comparison of clinical data between neonates screened as positive and negative.

Variable	Positive group	Negative group	*P*-value	*χ* ^2^	*Z*
Sex					
Male	343	72	0.379	0.772	
Female	338	60			
Age (days)^a^	4.0	4.0	0.358		−0.920
Birth weight (g)^a^	3,320.0	3,317.5	0.934		−0.082
Gestational age (weeks)^a^	39.0	39.0	0.106		−1.618
Maternal age (years)^a^	30.0	30.0	0.716		−0.364

^a^ Median, assessed using the rank sum test.

**Table 3:** Multivariate logistic regression results between neonates screened as positive and negative.

Variable	*β*	SE	Wald	*P*-value	OR	95% CI for OR
Constant	8.260	3.669	5.068	0.024	3,867.174	
Sex	0.198	0.193	1.052	0.305	1.219	0.835‒1.781
Age (days)	−0.022	0.066	0.113	0.736	0.978	0.860‒1.112
Birth weight (g)	0.000	0.000	1.102	0.294	1.000	1.000‒1.001
Gestational age (weeks)	−0.207	0.098	4.472	0.034	0.813	0.671‒0.985
Maternal age (years)	0.009	0.024	0.153	0.696	1.009	0.963‒1.057

SE, standard error; OR, odds ratio; CI, confidence interval.

### 3.3 Comparison of data between PFO-positive and PFO-negative neonates

Cases diagnosed by physicians as ‘PFO or small ASD' were classified as PFO in the analysis. Overall, 649 neonates had PFO, with a positive screening rate of 79.8%. No significant difference was observed in any of the data between the PFO-positive and PFO-negative neonates (all *P* > 0.05). Multivariate logistic regression analysis was applied to test whether neonatal age and weight, gestational age, and maternal age were independent influencing factors for PFO (*P* < 0.05, Table 4).

**Table 4:** Multivariate logistic regression results between PFO-positive and PFO-negative neonates.

Variable	β	SE	Wald	*P*-value	OR	95% CI for OR
Constant	7.047	3.348	4.431	0.035	1,149.603	
Sex	0.074	0.177	0.176	0.675	1.077	0.761–1.524
Age (days)	0.017	0.063	0.069	0.793	1.017	0.898–1.151
Birth weight (g)	0.000	0.000	1.447	0.229	1.000	1.000–1.001
Gestational age (weeks)	−0.172	0.089	3.700	0.054	0.842	0.707–1.003
Maternal age (years)	−0.004	0.022	0.043	0.835	0.996	0.954–1.038

PFO, patent foramen ovale; SE, standard error; OR, odds ratio; CI, confidence interval.

### 3.4 Comparison of data between PDA-positive and PDA-negative neonates

Eighty-seven newborns had PDA, yielding a positive screening rate of 10.7%. None of the neonatal clinical data conformed to normal distribution; therefore, the rank sum test was applied to compare the quantitative data between the groups. No significant differences were observed in any of the data between the PDA-positive and PDA-negative neonates (all *P* > 0.05). Multivariate logistic regression analysis was performed to screen whether neonatal age and weight, gestational age, and maternal age were independent influencing factors for PDA (*P* < 0.05). The results showed that neonatal birth weight was an independent influencing factor for PDA (OR = 1.001, *P* < 0.05), while the other indicators were not (all *P* > 0.05) (Table 5).

**Table 5:** Multivariate logistic regression results between PDA-positive and PDA-negative neonates.

Variable	β	SE	Wald	*P*-value	OR	95% CI for OR
Constant	0.934	4.356	0.046	0.830	2.545	
Sex	0.238	0.230	1.066	0.302	1.268	0.808–1.992
Age (days)	−0.157	0.101	2.430	0.119	0.854	0.701–1.041
Birth weight (g)	0.001	0.000	3.861	0.049	1.001	1.000–1.001
Gestational age (weeks)	−0.102	0.116	0.782	0.377	0.903	0.720–1.132
Maternal age (years)	−0.029	0.028	1.064	0.302	0.971	0.919–1.027

PDA, patent ductus arteriosus; SE, standard error; OR, odds ratio; CI, confidence interval.

### 3.5 Comparison of data between VSD-positive and VSD-negative neonates

Seven neonates had VSD, yielding a positive screening rate of 0.86%. No significant difference was observed in any of the clinical data between VSD-positive and VSD-negative neonates (all *P* > 0.05). Multivariate logistic regression analysis was performed to screen whether neonatal age and weight, gestational age, and maternal age were independent influencing factors for VSD (*P* < 0.05, Table 6).

**Table 6:** Multivariate logistic regression results between VSD-positive and VSD-negative neonates.

Variable	β	SE	Wald	*P*-value	OR	95% CI for OR
Constant	−11.986	14.288	0.704	0.402	0.000	
Sex	0.877	0.849	1.067	0.302	2.404	0.455–12.698
Age (days)	−0.034	0.288	0.014	0.907	0.967	0.550–1.699
Birth weight (g)	0.000	0.001	0.026	0.873	1.000	0.998–1.002
Gestational age (weeks)	0.067	0.376	0.032	0.858	1.070	0.512–2.235
Maternal age (years)	0.122	0.085	2.030	0.154	1.129	0.955–1.335

VSD, ventricular septal defect; SE, standard error; OR, odds ratio; CI, confidence interval.

## 4 Discussion

To the best of our knowledge, this study is the first to describe the results of neonatal Echo screening at a hospital in Hangzhou. Based on the results of the rank sum test and multiple regression analysis, we identified gestational age at delivery as an influencing factor for neonatal Echo abnormalities, with a smaller gestational age being associated with a higher incidence of abnormalities. This is consistent with the results of some previous studies (11, 12), which indicated that CHD is more prevalent in preterm compared to full-term infants.

CHD is a significant global problem in the field of paediatric health (13). Singh et al. (14) previously reported that 7–9 of every 1,000 live births had CHDs, with approximately 25% classified as severe CHD. CHD is the most common congenital disability in China. In children, disorders affecting the blood supply associated with body organs and tissues can lead to tissue hypoxia symptoms and serious complications such as heart failure (15). CHD may cause symptoms such as haemoptysis, syncope, and dyspnoea shortly following birth, while in severe cases, it can directly cause infant death. It is the primary cause of death due to congenital malformations in the first year after birth (16–19). In CHD screening, PFO, pulmonary hypertension, atrial septal aneurysm, ductus arteriosus aneurysm, aberrant subclavian artery, and PDA closure at 3 months after birth must all be screened out (9). Although they do not belong to the CHD category, they are crucial for the diagnosis of diseases in children (9). In our study, only one of the 813 newborns had complex CHD, and the probability was less than that of Singh et al. (14). The reason may be that the hospital involved in the study was a general hospital, not a professional maternity hospital. Some pregnant women found abnormalities during the prenatal examination and were referred to the maternity hospital, which may lead to biased results.

Echo can be performed using multiple anatomical windows or imaging views, including the subxiphoid area, precardiac area, and suprasternal fossa, providing clear data about the heart's position, heart structure, atrioventricular and valve positions, shape, and wall activity, as well as information about the heart chamber size and wall thickness. In patients with CHD, Echo can identify the location and size of the defect and indicate the distances with the superior and inferior vena cava and valve. Concurrently, the use of colour Doppler blood flow signals not only helps understand the blood flow velocity but also quantitatively detects reflux, shunt, and stenosis in vascular diseases. In addition, Echo has many advantages, including non-invasiveness, simplicity, rapidity, dynamic monitoring, repeatability of the operation, and high diagnosis rate, thereby facilitating early diagnosis of the disease and providing detailed parameter information for early clinical treatment, which has important clinical significance (20). Echo has also been widely used in clinical settings and has gradually become an important auxiliary examination method for CHD diagnosis; the importance of Echo in CHD screening has been well documented (21–23). The Echo abnormalities found in this study include PFO, ASD, PDA, VSD, valvular regurgitation, Eustachian valve, tricuspid valve displacement, atrial septal aneurysm, etc. Due to the high proportion of positive newborns in PFO, ASD, PDA and VSD, this study focuses on their analysis.

The highest positive screening rate in neonatal Echo abnormality in this study was attributable to PFO or small ASD. Most physicians diagnosed this type of abnormality as ‘PFO or small ASD’ rather than providing a specific diagnosis, as they are difficult to distinguish on ultrasound images in some cases. In the present study, we uniformly regarded all cases of PFO or small ASD as PFO for statistical analysis; this may partially explain why the proportion of PFO in our study far exceeds that in the baseline population. This is an unavoidable disadvantage of this study, which may exaggerate the prevalence of PFO. The neonatal foramen ovale is an important cardiac structure during the fetal period. As a physiological channel in the fetal period, it receives arterial blood from the placenta. The blood enters the foetus via the umbilical vein and flows from the right to the left atrium to maintain fetal blood circulation (24, 25). Following the establishment of respiration after birth, the pulmonary atrial pressure decreases and left atrial pressure increases, facilitating the functional closure of the oval valve and fossa, subsequently achieving anatomical maturation with growth and development. PFO can also occur during this transitional period. If the PFO does not close within 1 year of birth, intervention is not necessarily required. However, if it is not closed completely after 3 years of age, it is considered a congenital disease. Although this subset of children may be asymptomatic in the early stage, this condition can ultimately be severe and lead to complications such as atrial fibrillation, palpitations, and migraine, and even migraine and stroke; therefore, early examination and diagnosis of the disease are crucial. Children with PFO require continuous monitoring. After 3 years of age, interventional occlusion is required to manage the condition. ASD is a common congenital disease with a prevalence of approximately 2 cases per 1,000 live births. The spontaneous closure rate of ASD in the first year after birth is 4%–96%, while the most important predictor of spontaneous closure is the size of the defect, with smaller defects being more likely to close than larger defects (25, 26). Several indicators involved in this study have not previously been identified as influencing factors for PFO. Considering the high incidence of PFO in newborns, as well as the high rate of spontaneous closure, few studies have focused solely on investigating PFO in neonates. In future studies, we aim to conduct follow-up investigations in newborns with PFO to further examine the influencing factors for PFO closure. Follow-up usually includes repeat Echo examinations in view of the extensive recommendations (27, 28). Echo is the gold standard for the diagnostic evaluation of the haemodynamic significance of PDA (29, 30) and can further help to estimate the shunt volume (31). Asymptomatic persistent PFO and PDA have been shown to increase the risk of cryptogenic ischaemic stroke and chronic pulmonary hypertension compared with most symptomatic CHDs that can be subjected to surgical repair or percutaneous occlusion in early childhood or early adulthood, without any detectable early warning signal (32). Therefore, screening newborns for PDA can be the earliest indication of its risk, and it is necessary for monitoring and tracking the disease. This study's findings suggest that neonatal birth weight is an important influencing factor for PDA. At present, no relevant study has yet presented this view; however, some prior studies have suggested that the incidence of PDA is higher in premature infants than in full-term infants and is inversely proportional to gestational age at birth (25, 30). In premature infants, the time required for complete atresia is inversely proportional to gestational age at birth, while some blood vessels take months to years to close (29). In our study, gestational age at birth was not identified as an influencing factor for PDA; however, as none of the newborns had a gestational age younger than 28 weeks in our study, our findings align with the aforementioned conclusions. Furthermore, our study's results suggest that the risk of PDA in small-weight newborns is higher than that in normal-weight newborns and may also be indirectly related to gestational age. Future studies including multicentre data are warranted to verify the accuracy of this result. For patients with PFO, ASD, and PDA, we adopted a follow-up strategy, recommending that patients undergo Echo review at 3 months, 6 months, 1 year, and 2 years of age to assess disease development.

VSD is the most common non-cyanotic CHD, accounting for 37% of the children with CHD. There are four main types of VSD: peri-membranous, muscular, outlet, and inlet VSD (33). Pulse oximeter and Echo are non-invasive diagnostic tools for VSD (34). Echo remains the primary imaging modality for diagnosing and characterizing VSD. Echo is the most important clinical tool to help diagnose VSD. Although most VSDs have no clinical significance, or are self-closing, Doppler Echo and colour flow imaging can be used for the accurate anatomical and haemodynamic evaluation of VSD to determine whether surgery or transcatheter intervention is required (33). The frequency of spontaneous atresia of muscular VSD is higher and earlier than that of other types of VSD. Moreover, 80%–90% of isolated muscular VSDs spontaneously heal at 12 months (35). In the current study using the Chinese neonatal CHD database, the 2-year closure rate of ASD and VSD was approximately 90%. In the absence of any obvious clinical complications or growth arrest, newborns with ASD or VSD have a high chance of spontaneous defect closure without surgery or catheter-based intervention (32). VSD and ASD with large defects also require early surgical treatment to prevent complications, such as heart failure or pulmonary hypertension. Riko et al. previously recommended treatment for premature infants with VSD larger than 2 mm, including surgical intervention, in collaboration with paediatric cardiologists (36). Several indicators included in our study were not identified as influencing factors for VSD. Therefore, we plan to conduct follow-up studies on children with VSD to explore additional insights and present further ideas.

This study has several limitations. First, the study findings are specific to a tertiary hospital in the eastern city of Hangzhou, China, and thus may not be generalisable to populations of other ethnicities or regions. Second, the Echo results may be affected by subjective factors, leading to variability in results. Third, other diagnostic methods, such as pulse oximetry, may also be used to screen for CHD; however, these were not discussed in this study. Finally, some pregnant women with abnormal prenatal examination results may have sought care at other specialised hospitals, potentially introducing bias in patient selection.

In conclusion, the present study found that the positive screening rate is very high in Echo screening. While most newborns have the capacity to heal autonomously, early detection of abnormalities and continuous monitoring remain crucial. This study highlights the importance of timely pregnancy testing for early pregnancy detection and prenatal care, potentially mitigating the risk of premature birth and low birth weight. Notably, neonatal Echo screening is not universally practiced across all cites in China. Echo promotes early detection and early diagnosis of CHD. This study could prompt increased attention from paediatricians towards Echo findings and have positive significance for the promotion of national neonatal Echo census. And we aim to continue performing follow-up studies on abnormal newborns to promote further discussion.

## Data Availability

The raw data supporting the conclusions of this article will be made available by the authors, without undue reservation.
